# Community-acquired and hospital-acquired bacterial co-infections in patients hospitalized with Covid-19 or influenza: a retrospective cohort study

**DOI:** 10.1007/s15010-023-02063-2

**Published:** 2023-06-16

**Authors:** Anselm Jorda, Cornelia Gabler, Amelie Blaschke, Michael Wölfl-Duchek, Georg Gelbenegger, Alina Nussbaumer-Pröll, Christine Radtke, Markus Zeitlinger, Felix Bergmann

**Affiliations:** 1 Department of Clinical Pharmacology, Medical University of Vienna, Währinger Gürtel 18-20, 1090 Vienna, Austria; 2 IT Systems and Communications, Medical University of Vienna, Vienna, Austria; 3 Division of Infectious Diseases and Tropical Medicine, Department of Medicine I, Medical University of Vienna, Vienna, Austria; 4 Department of Biomedical Imaging and Image-Guided Therapy, Medical University of Vienna, Vienna, Austria; 5 Department of Plastic, Reconstructive and Aesthetic Surgery, Medical University of Vienna, Währinger Gürtel 18-20, 1090 Vienna, Austria

**Keywords:** SARS-CoV-2, Pneumonia, Bacterial superinfection, Coronavirus, Flu, Noncritical, Ward

## Abstract

**Background:**

Bacterial co-infections are believed to be less frequent in patients with Covid-19 than influenza, but frequencies varied between studies.

**Methods:**

This single-center retrospective, propensity score-matched analysis included adult patients with Covid-19 or influenza admitted to normal-care wards between 02/2014 and 12/2021. Covid-19 cases were propensity score matched to influenza cases at a 2:1 ratio. Community-acquired and hospital-acquired bacterial co-infections were defined as positive blood or respiratory cultures ≤ 48 h or > 48 h after hospital admission, respectively. The primary outcome was comparison of community-acquired and hospital-acquired bacterial infections between patients with Covid-19 and influenza in the propensity score-matched cohort. Secondary outcomes included frequency of early and late microbiological testing.

**Results:**

A total of 1337 patients were included in the overall analysis, of which 360 patients with Covid-19 were matched to 180 patients with influenza. Early (≤ 48 h) microbiological sampling was performed in 138 (38.3%) patients with Covid-19 and 75 (41.7%) patients with influenza. Community-acquired bacterial co-infections were found in 14 (3.9%) of 360 patients with Covid-19 and 7 (3.9%) of 180 patients with influenza (OR 1.0, 95% CI 0.3–2.7). Late (> 48 h) microbiological sampling was performed in 129 (35.8%) patients with Covid-19 and 74 (41.1%) patients with influenza. Hospital-acquired bacterial co-infections were found in 40 (11.1%) of 360 patients with Covid-19 and 20 (11.1%) of 180 patients with influenza (OR 1.0, 95% CI 0.5–1.8).

**Conclusion:**

The rate of community-acquired and hospital-acquired bacterial co-infections was similar in hospitalized Covid-19 and influenza patients. These findings contrast previous literature reporting that bacterial co-infections are less common in Covid-19 than influenza.

**Supplementary Information:**

The online version contains supplementary material available at 10.1007/s15010-023-02063-2.

## Introduction

Bacterial co-infections with concurrent viral infections can exacerbate disease and prolong hospitalization [1, 2]. Preceding or concurrent viral infections are thought to predispose patients to secondary bacterial co-infections. Although the underlying mechanisms are complex and have not yet been fully elucidated, viral infections are thought to trigger a cascade of host immune responses that favorably alter microbial growth in the upper and lower respiratory tract and gut [3, 4]. Previous studies have investigated the prevalence of community-acquired and hospital-acquired bacterial co-infections in Covid-19 and influenza, their results were highly inconsistent. In a systematic review by Klein et al., bacterial co-infections were observed in 2–65% of influenza patients, whereas early bacterial infections amounted to 1.2–8% in studies of Covid-19 patients [5–7]. These heterogeneous results may be attributed to geographic and temporal differences among studies, insufficient information on test methods for bacterial detection, inadequate distinction between contamination or colonization and infection, and inconsistent definitions of community-acquired and hospital-acquired infection [8]. This uncertainty presents a challenge for physicians in managing early antimicrobial therapy.

Current guidelines on the antimicrobial treatment and management of Covid-19 advise against the use of empiric antibiotic therapy in the absence of proven or suspected bacterial infection [9]. Although bacterial co-infections have been shown to be rather rare, the lack of distinguishing features between pneumonia of viral or bacterial origin may obscure their true prevalence. Nevertheless, the use of empiric antimicrobial treatment remains high and unstandardized [10, 11]. Therefore, the aim of this study was to describe the prevalence and microbiological spectrum of community-acquired (identified within 48 h of admission) and hospital-acquired (identified after 48 h of admission) bacterial co-infections in patients admitted to normal care wards with Covid-19 or influenza.

## Methods

### Study design and setting

This retrospective observational cohort study was conducted at the Medical University of Vienna, Austria and received ethics approval from the local Ethics Committee before initiation (EC 2259/2021). Data were collected by automated extraction from electronic medical records. Incomplete and implausible data were manually reviewed and refined. This observational study was performed in accordance with the STROBE (Strengthening the Reporting of Observational Studies in Epidemiology) recommendations [12].

### Study population

We collected data from patients hospitalized with either Covid-19 or influenza (A or B), confirmed by a positive polymerase chain reaction (PCR) test during hospitalization. The automatic case extraction of both groups included patient data from February 2014 to December 2021. Eligibility criteria included age ≥ 18 years and admission to a normal care ward. Patients were excluded if they were directly admitted to the intensive care unit (ICU) or transferred from another institution to avoid temporal misclassification of bacterial infections.

### Microbiological definitions

Microbiological findings were interpreted as bacterial respiratory co-infections if cultures from respiratory or blood samples or a urinary antigen test for *Legionella pneumophila* or *Streptococcus pneumoniae* yielded positive results [13]. Respiratory sampling included bronchoalveolar lavage samples, endotracheal aspirates and sputum. Clinically insignificant findings (i.e., commensal skin bacteria and staphylococci other than *Staphylococcus aureus)* were excluded*.* Furthermore, fungal pathogens were excluded from the analysis. Table S1 provides the complete list of excluded pathogens with respective explanations. Community-acquired infection was defined as a positive culture of one or more pathogens within 48 h of hospital admission. Positive samples collected after 48 h of admission were interpreted as evidence of hospital-acquired infections [14]. When patients with community-acquired infections yielded positive results with new pathogens during microbiologic sampling 48 h after admission, they were included in the analysis of both community-acquired and hospital-acquired infections.

### Outcome parameters

The primary outcomes of this study were comparison of community-acquired and hospital-acquired bacterial infections between patients with Covid-19 and influenza in the propensity score-matched cohort. Secondary outcomes included the comparison of community-acquired and hospital-acquired bacterial infections between patients with Covid-19 and influenza in the overall (i.e., unmatched) cohort, prevalence and distribution of identified bacterial pathogens, results from routine antimicrobial susceptibility testing (AST) according to the European Committee on Antimicrobial Susceptibility Testing (EUCAST), time to bacterial co-infection and 28-day all-cause mortality. Considering the risk of detection bias, we additionally evaluated early (≤ 48 h) and late (> 48 h) microbiologic sampling (blood and/or respiratory) frequencies in both groups.

### Statistical analysis

Baseline characteristics are reported descriptively with mean ± standard deviation (SD) or numbers (%) and were compared using standardized mean differences. The frequency of community- and hospital-acquired bacterial co-infections between matched and unmatched groups were compared with the Fisher’s exact test. Pathogen distribution and prevalence were reported descriptively. To assess the correlation between the occurrence of bacterial co-infections and the biochemical inflammatory response, we compared the highest C-reactive protein (CRP) and procalcitonin levels between patients with and without bacterial co-infections using boxplots and the independent sample t-test. We performed a pooled analysis of early and late as well as Covid-19 and influenza patients. Sensitivity analyses included the comparisons of sampling frequencies and co-infection rates in both groups over time and between the Covid-19 variants. The statistical analysis and visualization were performed using R (Version 2021.09.2) and GraphPad Prism 9.3.1.

### Propensity score matching

To adjust for baseline differences between the Covid-19 and influenza group, we performed propensity score matching using the R package ‘MatchIt’ (Version 4.4.0). Because the number of available Covid-19 cases exceeded that of influenza cases, Covid-19 cases were matched to influenza cases at a 2:1 ratio with the optimal pair matching method. Propensity scores were estimated by logistic regression. We modeled the viral disease (Covid-19 or influenza) as the dependent variable and covariates as independent variables (Table 1). We assessed the group balance using standardized mean differences, with values > 0.1 indicating potentially significant differences. The included covariates were selected to minimize the standardized mean differences and included age, sex, length of hospital stay, relevant baseline diseases (diabetes mellitus, coronary artery disease, chronic heart failure, chronic obstructive pulmonary disease, and chronic kidney disease), and the frequency of early (≤ 48 h) and late (> 48 h) microbiological testing.

**Table 1:** Baseline characteristics of the overall and matched study cohorts

	Overall cohort			Matched cohort		
	Covid-19 (*n* = 1157)	Influenza (*n* = 180)	Standardized difference	Covid-19 (*n* = 360)	Influenza (*n* = 180)	Standardized difference
Demographic characteristics						
Male sex, *n* (%)	612 (52.9)	94 (52.2)	0.013	176 (48.9)	94 (52.2)	0.07
Age, mean (SD)	58.9 (19.3)	60.2 (19.0)	0.065	58.6 (19.0)	60.2 (19.0)	0.09
Length of hospital stay (days), mean (SD)	16.4 (21.4)	22.5 (27.4)	0.246	20.6 (27.8)	22.5 (27.4)	0.07
Baseline comorbidities, *n* (%)						
Diabetes	210 (18.2)	42 (23.3)	0.128	84 (23.3)	42 (23.3)	< 0.001
Coronary artery disease	187 (16.2)	50 (27.8)	0.283	94 (26.1)	50 (27.8)	0.04
Chronic heart failure	50 (4.3)	16 (8.9)	0.185	28 (7.8)	16 (8.9)	0.04
Asthma	25 (2.2)	5 (2.8)	0.04	9 (2.5)	5 (2.8)	0.02
Chronic obstructive pulmonary disease	76 (6.6)	27 (15.0)	0.274	52 (14.4)	27 (15.0)	0.02
Hypertension	436 (37.7)	88 (48.9)	0.228	165 (45.8)	88 (48.9)	0.06
Chronic kidney disease	128 (11.1)	47 (26.1)	0.394	90 (25.0)	47 (26.1)	0.03
Skin disorder	105 (9.1)	24 (13.3)	0.135	50 (13.9)	24 (13.3)	0.02
Mental disorder	238 (20.6)	44 (24.4)	0.093	93 (25.8)	44 (24.4)	0.03
Neurologic disorder	202 (17.5)	45 (25.0)	0.185	78 (21.7)	45 (25.0)	0.079
Microbiological sampling, *n* (%)						
Early culture available (≤ 48 h)	357 (30.9)	75 (41.7)	0.226	138 (38.3)	75 (41.7)	0.068
Late culture available (< 48 h)	323 (27.9)	74 (41.1)	0.28	129 (35.8)	74 (41.1)	0.10

### Sample size

The results of previous studies investigating the prevalence of community-acquired and hospital-acquired bacterial co-infections in both Covid-19 and influenza remain highly heterogenous. Nevertheless, co-infections are thought to be more common with influenza than with Covid-19, both in patients receiving normal treatment and intensive care [15]. We estimated a risk difference of approximately 10% to be a clinically important difference (5% vs. 15%) and calculated the sample size accordingly. We performed matching at a 2:1 ratio due to a higher number of Covid-19 than influenza cases to obtain a higher statistical power while preventing the inclusion of influenza cases, whose observation period was too far in the past. As a result, a sample size of 540 cases matched at a 2:1 ratio (360 Covid-19 cases vs 180 influenza cases) provided a 95% power to detect a statistically significant group difference of 10% with the Fisher’s exact test at a two-sided alpha of 0.05. We included influenza cases in reverse chronological order starting from December 2021 until we reached the desired sample of 180 evaluable cases (February 2014). All Covid-19 cases were included until December 2021.

## Results

### Study population before and after propensity score matching

**Fig. 1:**
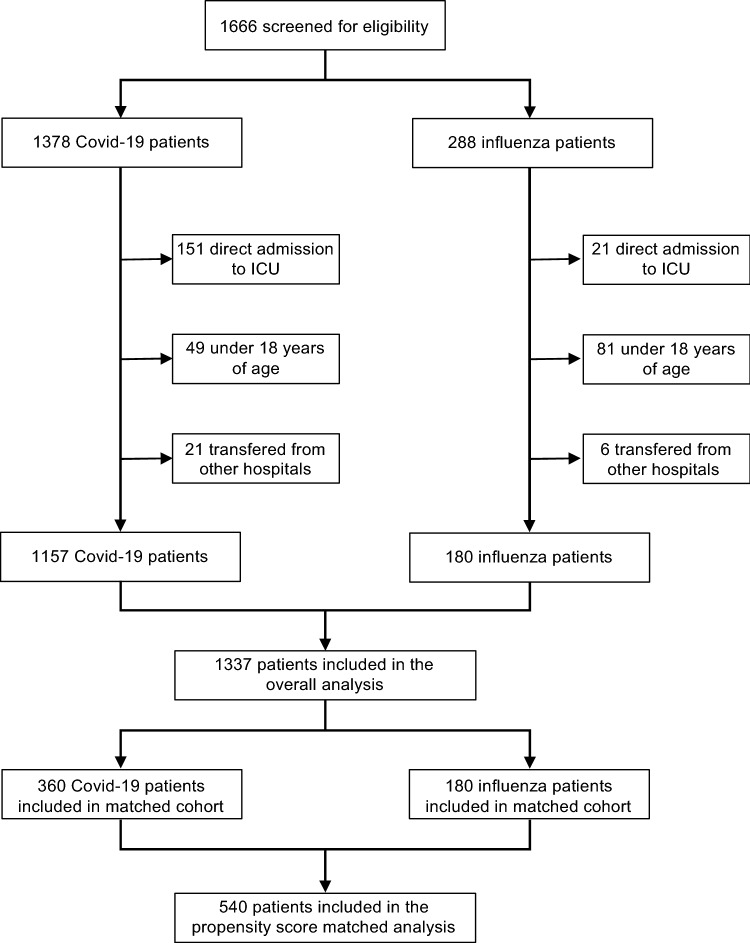
Flow chart of the study population

To obtain the prespecified sample size of 180 eligible patients with influenza, we screened a total of 288 patients with influenza in reverse chronological order starting from December 2021 to February 2014 (Fig. 1 and Fig. S1). A total of 1378 patients with Covid-19, who were admitted between January 2020 and December 2021, were identified. After excluding patients transferred from another institution (21 Covid-19 cases and 6 influenza cases), patients aged below 18 (49 Covid-19 cases and 81 influenza cases), and patients admitted directly to an intensive care unit (151 Covid-19 cases and 21 influenza cases), the final pre-matching analysis set comprised 1157 patients with Covid-19 and 180 patients with influenza.

Table 1 provides the baseline characteristics of both groups. In the overall cohort, we observed meaningful group differences, as indicated by standardized differences > 0.1, in the covariates length of hospital stay and in the prevalence of diabetes, coronary artery disease, chronic heart failure, chronic obstructive pulmonary disease, skin disorders, and neurological disorders. Before matching, the prevalence of baseline comorbidities was generally lower in the Covid-19 group than in the influenza group. Moreover, the frequency of early and late testing was lower in the Covid-19 group than in the influenza group (Table 1).

Of the 180 influenza cases, 134 (74.4%) were caused by influenza virus A and 46 (25.6%) were caused by influenza virus B (Fig. S2). Of the 1157 Covid-19 cases, 192 (16.6%) were caused by the wildtype virus, 671 (58.0%) by the alpha variant, 245 (21.2%) by the delta variant, and 49 (4.2%) cases were not sequenced (Fig. S3).

We matched 360 patients with Covid-19 to 180 patients with influenza (ratio 2:1). As shown in Table 1, propensity score matching resulted in a good balance of all covariates between the two groups (standardized mean differences ≤ 0.1).

### Microbiological findings in the propensity score matched cohort

**Fig. 2:**
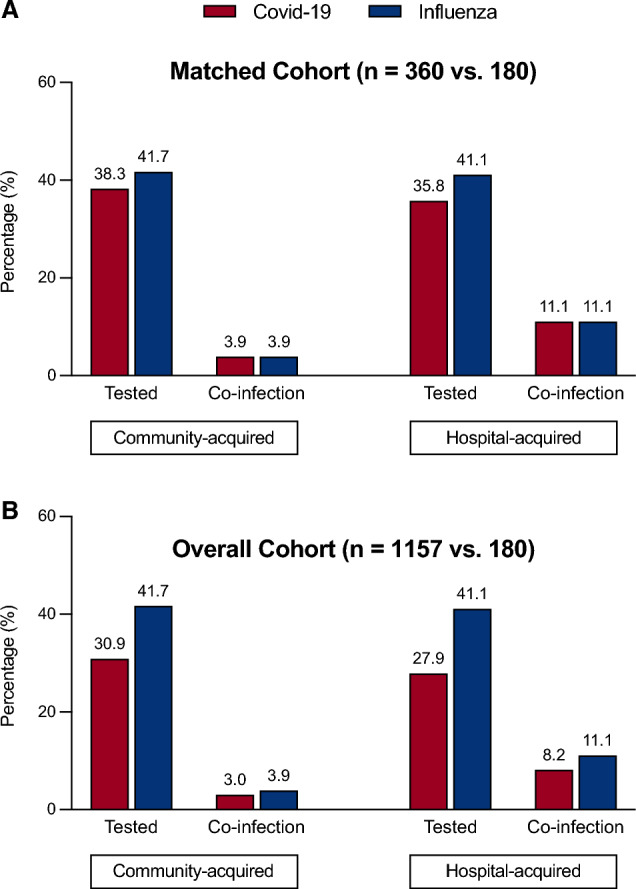
Microbiological testing frequencies and identification of bacterial pathogens in the **A** propensity score-matched cohort and **B** overall cohort. Community-acquired co-infections included microbiological tests performed within 48 h of hospital admission. Hospital-acquired co-infections included microbiological tests performed after 48 h of hospital admission

**Fig. 3:**
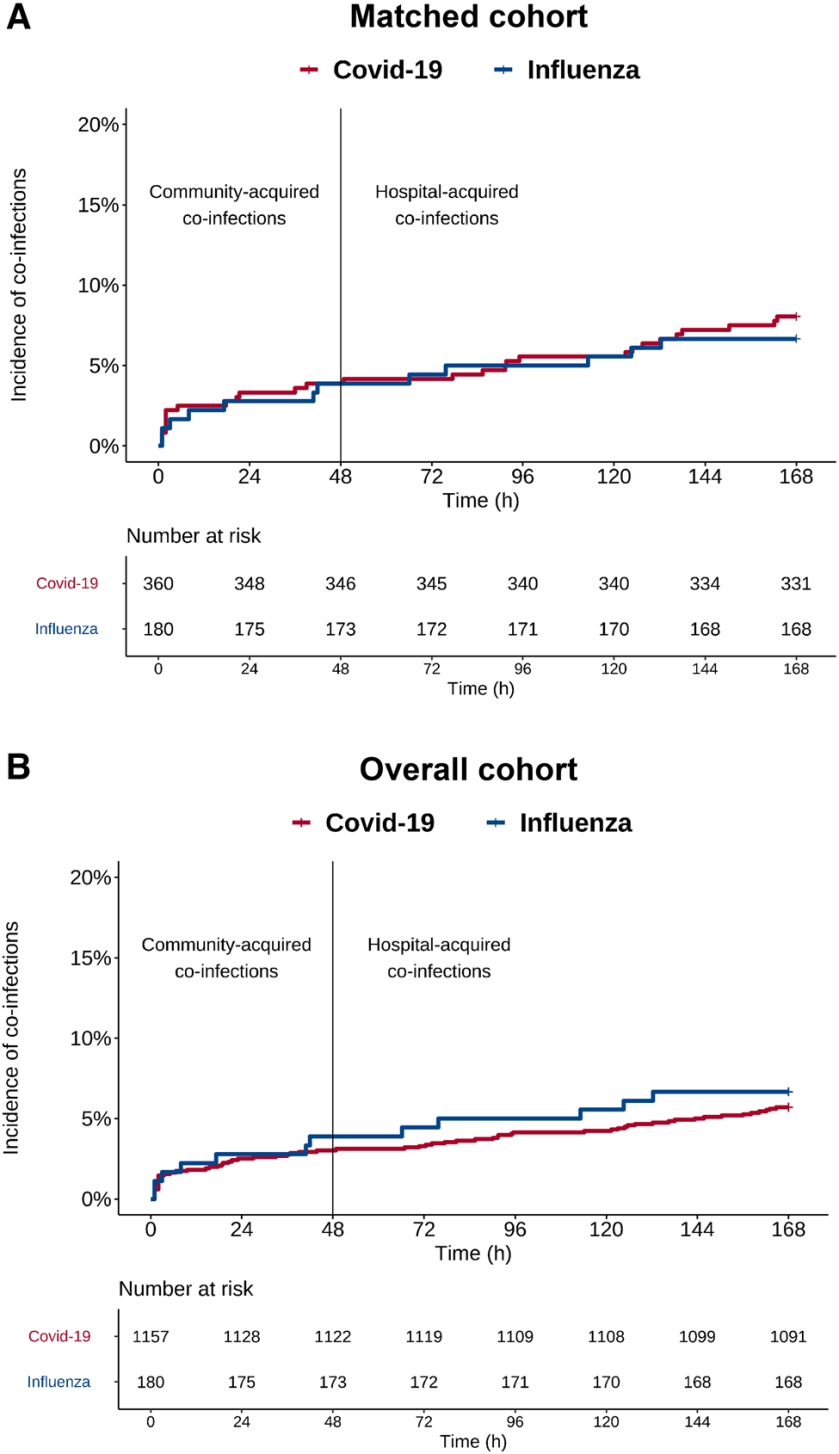
Time to bacterial co-infection. Group comparison of time to first identification of bacterial pathogen in hours (h) after hospital admission in the **A** propensity score-matched cohort and **B** overall cohort

In the propensity score-matched cohort, early (≤ 48 h) microbiological sampling (respiratory and/or blood) was performed in 138 (38.3%) of 360 patients with Covid-19 and 75 (41.7%) of 180 patients with influenza. The relative frequency of community-acquired bacterial co-infections was identical between the two matched groups and was found in 14 (3.9%) of 360 patients with Covid-19 and 7 (3.9%) of 180 patients with influenza (OR 1.0, 95% CI 0.34–2.7, *p* = 1.0) (Fig. 2A). Late (> 48 h) microbiological sampling (respiratory and/or blood) was performed in 129 (35.8%) of 360 patients with Covid-19 and 74 (41.1%) of 180 patients with influenza. The relative frequency of hospital-acquired bacterial co-infections was identical between the two matched groups and was found in 40 (11.1%) of 360 patients with Covid-19 and 20 (11.1%) of 180 patients with influenza (OR 1.0, 95% CI 0.54–1.82, *p* = 1.0) (Fig. 2A). Figure 3 depicts the time to bacterial co-infection in both cohorts. Bacterial co-infections were predominantly confirmed in respiratory samples. Blood cultures were positive in 1 of the 14 cases of Covid-19 patients with community-acquired infections and in 4 of the 40 cases of Covid-19 patients with hospital-acquired infections. No positive blood cultures were identified in influenza patients.

### Microbiological findings in the overall cohort

In the overall cohort, early microbiological sampling was performed significantly less often in patients with Covid-19 (357 [30.9%] of 1157) than in patients with influenza (75 ([41.7%] of 180) (OR 0.6, 95% CI 0.45–0.88, *p* = 0.005). The relative frequency of community-acquired bacterial co-infections was similar between the two unmatched groups and was found in 35 (3.0%) of 1157 patients with Covid-19 and 7 (3.9%) of 180 patients with influenza (Fig. 2B). Late sampling was performed less often in Covid-19 patients (323 [27.9%]) of 1157) than in influenza patients (74 [41.1%] of 180) (OR 0.55, 95% CI 0.4–0.78, *p* = 0.0004). The relative frequency of hospital-acquired bacterial co-infections was numerically lower in patients with Covid-19 (95 [8.2%] of 1157) than in patients with influenza (20 [11.1%] of 180) (Fig. 2B).

Blood cultures were positive in 2 of the 35 cases of Covid-19 patients with community-acquired infections and in 6 of the 95 cases of Covid-19 patients with hospital-acquired infections. No positive blood cultures were identified in influenza patients.

### Identified pathogens

**Fig. 4:**
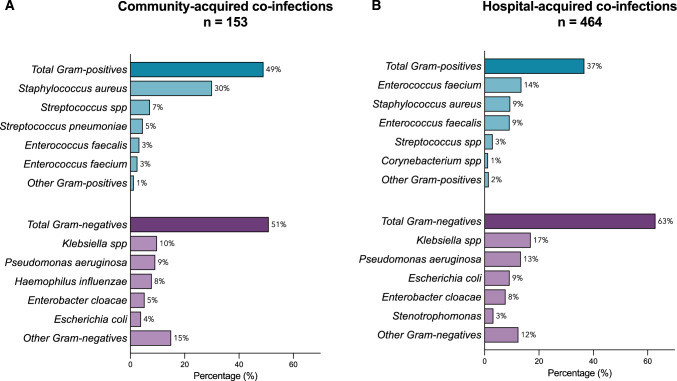
Relative frequencies of **A** early (≤ 48 h) and **B** late (> 48 h) identified Gram-positive and Gram-negative pathogens. Due to the low number of identified pathogens in the influenza group, data from Covid-19 and influenza patients were pooled

In the overall cohort, a total of 153 (75 [49.0%] Gram-positive and 78 [51.0%] Gram-negative) isolates were identified as causative pathogens of community-acquired infections (Fig. 4). The most common early identified Gram-positive pathogens were *S. aureus* (46 [30.0%] of 153), *Streptococcus* spp. other than *S. pneumoniae* (11 [7.2%] of 153), and *S. pneumoniae* (7 [4.6%] of 153). The most common early identified Gram-negative pathogens were *Klebsiella* spp. (15 [9.8%] of 153), *P. aeruginosa* (14 [9.2%] of 153), and *H. influenzae* (7 [4.6%] of 153).

A total of 464 (171 [36.9%] Gram-positive and 293 [63.1%] Gram-negative) isolates were identified as causative pathogens of hospital-acquired infections. The most common late identified Gram-positive pathogens were *E. faecium* (63 [13.6%] of 464), *S. aureus* (44 [9.5%] of 464), and *E. faecalis* (43 [9.3%] of 464). The most common late identified Gram-negative pathogens were *Klebsiella* spp. (79 [17.0%] of 464), *P. aeruginosa* (62 [13.4%] of 464), and *E. coli* (43 [9.3%] of 464).

The proportion of Gram-negative pathogens was significantly higher in hospital-acquired infections than in community-acquired infections (293 [63.1%] of 464 vs 78 [50.9%] of 153, OR 1.65, 95% CI 1.12–2.43, *p* = 0.01).

### Resistance of identified pathogens

**Fig. 5:**
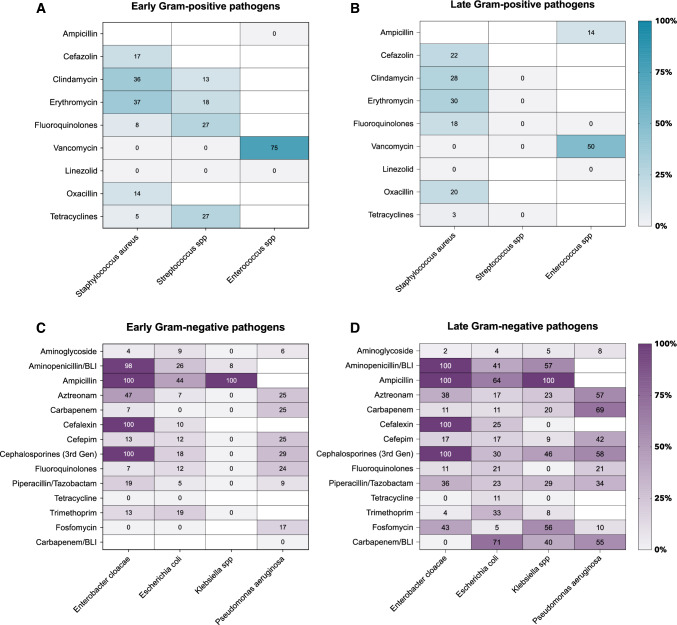
Antimicrobial resistance of identified pathogens. Relative frequency (%) of antimicrobial resistance of identified pathogens as assessed by routine susceptibility testing. **A** Early and **B** late identified Gram-positive pathogens and their resistance to tested antibiotics. **C** Early and **D** late identified Gram-negative pathogens and their resistance to tested antibiotics. Of note, not all isolates were tested against all antibiotics. Hence, denominators in the resulting percentages may differ. Empty fields indicate that no tests have been performed for the selected pair. Aminopenicillin/beta-lactamase inhibitors (BLI) included amoxicillin/clavulanic acid and ampicillin sulbactam. Carbapenem/BLI included meropenem/vaborbactam and imipenem/relebactam. 3rd generation cephalosporines included cefixime, ceftriaxone, cefotaxime, and ceftazidime

Figure 5 shows the relative frequency of antimicrobial resistance, as assessed by routine susceptibility testing according to EUCAST, of the most frequently identified pathogens. Importantly, not all isolates were tested against all antibiotics, which may have skewed the observed frequency of resistance. Overall, the percentage of antimicrobial resistance was higher in Gram-negative isolates causing hospital-acquired infections than those causing community-acquired infections. This visual trend was not observed for Gram-positive pathogens.

### Mortality

In the matched cohort, the 28-days all-cause mortality was significantly higher in Covid-19 than influenza patients (39 [10.8%] of 360 vs. 7 [3.9%] of 180, OR 3.0, 95% CI 1.3–8.1, *p* = 0.005) (Fig. S4). Similarly, in the unmatched cohort, the 28-days all-cause mortality was significantly higher in Covid-19 than influenza patients (123 [10.6%] of 1157 vs. 7 [3.9%] of 180, OR 2.9, 95% CI 1.4–7.6, *p* = 0.003) (Fig. S5). In the overall Covid-19 group, community-acquired co-infections were significantly associated with an increased 28-day mortality (8 [22.9%] of 35 vs. 115 [10.3%] of 1122, OR 2.6, 95% CI 1.2–6.0, *p* = 0.026). This association could not be observed in the propensity score matched Covid-19 group or in the influenza groups, due to the limited sample size (data not shown).

### Inflammatory response

Inflammatory markers were higher in patients with bacterial co-infections. CRP levels were 26.1 ± 11.1 mg/dL in the co-infection group and 10.0 ± 10.1 mg/dL in the group without co-infections (*p* < 0.001) (Fig. S6). Procalcitonin levels were 6.9 ± 15.4 ng/L in the co-infection group and 1.8 ± 7.4 mg/dL in the group without co-infection (*p* < 0.001) (Fig. S7).

### Subgroup and sensitivity analyses

**Fig. 6:**
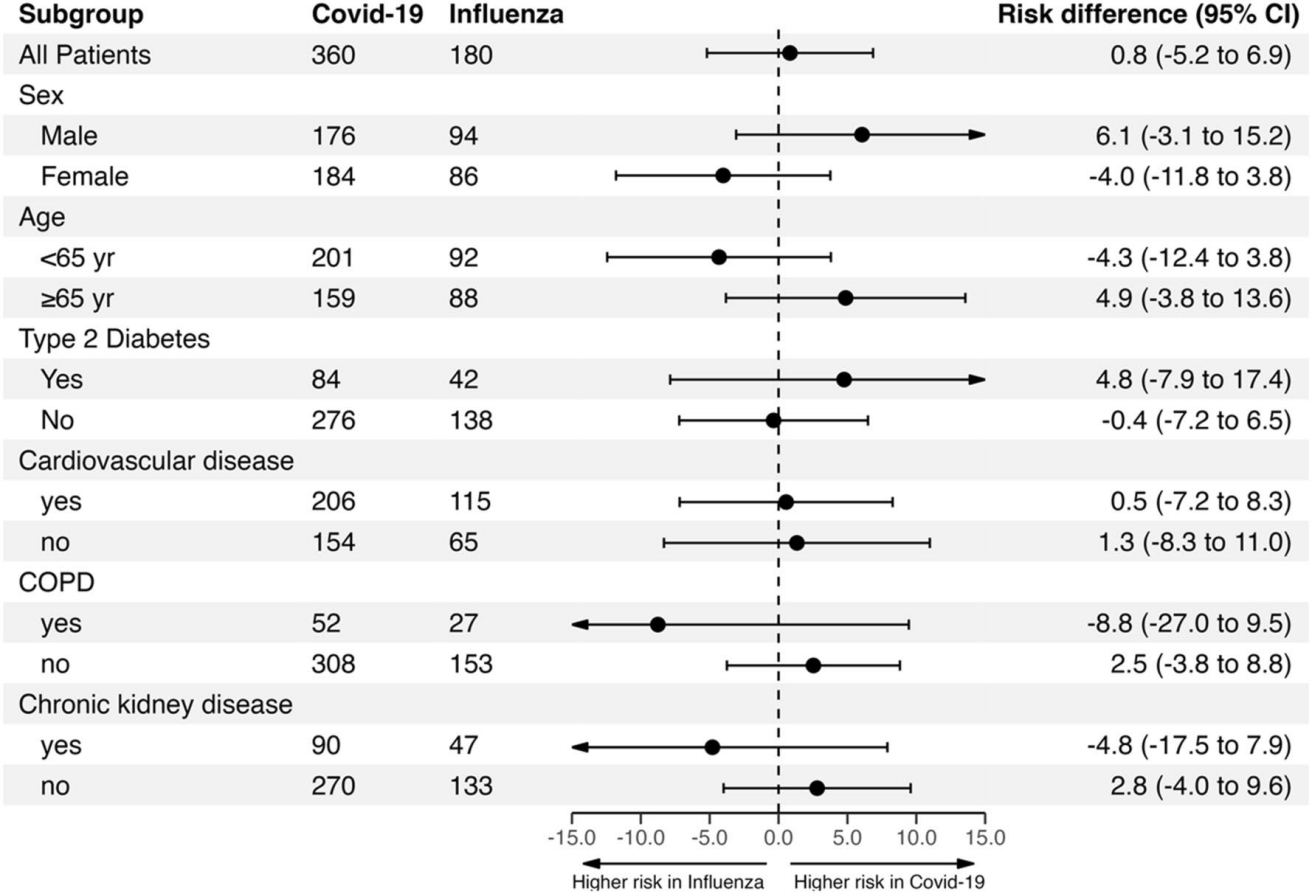
Estimated risk difference of developing any bacterial co-infection (identification ≤ 48 h and/or > 48 h of admission) in subgroup analyses

Subgroup analyses revealed no significant group differences in the risk of developing any bacterial co-infections (identification ≤ 48 h or > 48 h of admission) (Fig. 6). Moreover, we observed constant rates of microbiological testing, identification of bacterial co-infection, ICU admission, and mortality over time in both groups (Figs. S8 and S9). In the Covid-19 group, early bacterial co-infections were most common in patients with the delta variant (14 [5.7%] of 245) and least common in patients with the wildtype SARS-CoV-2 virus (3 [1.6%] of 192) (Table S2).

## Discussion

The present study compared community-acquired and hospital-acquired bacterial co-infections between hospitalized Covid-19 and influenza patients. According to previous literature, bacterial co-infections are more common in influenza than in Covid-19, both in patients receiving standard level and intensive care [15]. However, our data demonstrate a similar rate of community-acquired bacterial co-infections in the matched Covid-19 (4%) and influenza (4%) groups. In patients with Covid-19, community-acquired co-infections were rare but significantly associated with an increased 28-days all-cause mortality. Hospital-acquired infections were also similar between Covid-19 (11%) and influenza (11%).

In the unmatched analysis, patients with Covid-19 displayed a numerically lower prevalence of community-acquired (3% vs. 4%) and hospital-acquired (8% vs. 11%) co-infections than patients with influenza. However, before matching, the frequency of microbiological sampling was significantly lower in Covid-19 than influenza patients for both community-acquired (31% vs. 42%) and hospital-acquired (28% vs. 41%) infections, which may explain the difference in co-infection rates between the two groups. The lower number of samples collected in Covid-19 may be due to more stringent safety precautions in treating Covid-19 patients and limited resources, especially in the early phases of the pandemic. In both matched and overall cohorts, community-acquired co-infections were found in about 10% of patients who were tested within 48 h of hospital admission; hospital-acquired co-infections were found in about 25% of patients who were tested after 48 h of admission. It is likely that a higher sampling frequency would have resulted in a higher incidence of co-infections.

Previous studies have examined the prevalence of bacterial co-infections in both groups, with results ranging from 2 to 65% and 1.2 to 8% in influenza and Covid-19 patients, respectively [5–7]. This disparity may be attributed to underlying methodological heterogeneity between studies. First, many analyses did not consider differences in patient characteristics when comparing the two groups. Our results showed that hospitalized influenza patients were, on average, older and had more comorbidities than Covid-19 patients. In addition, microbiological testing was performed less frequently in Covid-19 patients. Thus, the resulting difference in bacterial co-infections may be skewed when comparing unmatched cohorts, which may explain the higher prevalence of co-infections in influenza patients documented in literature. Second, most studies provided only vague definitions of significant positive microbiological specimens that inadequately described the pathogens considered to be associated with the infection or were based solely on clinical significance as determined by the treating physicians [16, 17]. However, due to limited distinguishing diagnostic features between viral and bacterial infections, clinical significance may have been incorrectly determined. In Table S1, we provide an overview of the pathogens that we excluded from our analysis, along with the justification for their exclusion. Third, some studies considered pathogens to be associated with community-acquired or hospital-acquired infections that were collected at times other than ≤ 48 h or > 48 h of admission, respectively [6, 11, 18]. Finally, as addressed by Thaden et al., pooling these studies in meta-analyses is problematic due to heterogenous study designs and microbiological definitions [8, 19–21]. Nevertheless, data on bacterial co-infections from different geographical regions are important to account for the variability in differences in testing practices, the microbiology of bacterial infections or antimicrobial stewardship policies [8].

Overall, most common Gram-positive and Gram-negative pathogens isolated causing community-acquired co-infections were *S. aureus*, *Streptococcus* spp, *S. pneumoniae*, and *Klebsiella* spp., *P. aeruginosa, H. influenzae*, respectively. In contrast, most common Gram-positive and Gram-negative pathogens causing hospital-acquired infections included *E. faecium*, *S. aureus*, *E. faecalis* and *Klebsiella* spp., *P. aeruginosa, E. coli*, respectively. These identified pathogens are well in line with previous literature [13, 15, 17, 22]. Although respiratory tract infections with Enterococcus spp. are considered rare, previous reports have shown that their occurrence can complicate treatment and their incidence may be underestimated [23]. We observed a visual increase in antimicrobial resistance between community-acquired and hospital-acquired Gram-negative pathogens. These findings are consistent with previous reports that antimicrobial resistance is associated with prolonged hospital stay [24]. This trend was not observed in Gram-positive pathogens. However, not all pathogens were routinely tested against all antibiotics, which may skew the true rate of antimicrobial resistance.

We acknowledge several limitations of this study. First, medication and antibiotic use were not available in our data set. Nevertheless, adjustment for antibiotic use is challenging due to the multifactorial heterogeneity of prescribed substances, underlying pathogens, prescription timing and treatment duration. In addition, including antibiotic use as a covariate into the analyses carries the risk of reverse causality. On the one hand, patients without antimicrobial treatment may be at higher risk for co-infections, on the other hand, empirical antimicrobial therapy may be given more often in patients at risk. Second, Covid-19 and influenza patients were not hospitalized contemporaneously. However, the single center study design may have contributed to minimizing differences in treatment practices in both groups. Moreover, our sensitivity analyses did not reveal changes in the outcomes of influenza patients over time, suggesting that the comparison between the two groups is valid. Third, our sample size calculation was designed to provide a sufficient statistical power to detect clinically important group differences (i.e., a risk difference of 10%) in the rate of bacterial co-infections, which we did not observe. However, this study was not powered to detect more subtle group differences, which cannot be ruled out based on our findings. Fourth, our analysis excluded patients who were admitted directly to an intensive care unit. Therefore, the incidence of bacterial co-infection could be higher in all patients, regardless of the type of initial admission, due to the higher incidence in critically ill patients [25, 26]. Finally, the correct classification of clinically relevant bacterial co-infections remains challenging. However, by strictly adhering to the definitions of bacterial co-infections and transparently listing the included and excluded pathogens, we believe that our results provide a sound comparison between the two patient groups.

## Conclusion

The prevalence of community-acquired and hospital-acquired bacterial co-infections was similar in hospitalized Covid-19 and influenza patients admitted to normal care wards. These findings contrast previous literature reporting that bacterial co-infections are less common in Covid-19 than influenza.

### Supplementary Information

Below is the link to the electronic supplementary material.

Supplementary file1 (DOCX 2931 KB)

## Data Availability

The data and materials that support the findings of this study are available from the corresponding author upon the reasonable request.
