# Supplementary material for: Community-acquired and hospital-acquired bacterial co-infections in patients hospitalized with Covid-19 or influenza: a retrospective cohort study
Source: Infection. 2023 Jun 16;52(1):105–15. doi: 10.1007/s15010-023-02063-2 (PMC10811098; doi:10.1007/s15010-023-02063-2)
Supplement: Supplementary file 1 — Supplementary file1 (DOCX 2931 KB) [file 15010_2023_2063_MOESM1_ESM.docx]

**Supplement**

**Community-acquired and hospital-acquired bacterial co-infections in patients admitted to normal care wards with Covid-19 or Influenza: a retrospective cohort study**

**Running Title:**

**Bacterial Co-infections in Covid-19 and Influenza**

**Authors:**

Anselm Jorda^1^, Cornelia Gabler^2^, Amelie Blaschke^1,3^, Michael Wölfl-Duchek^1,4^, Georg Gelbenegger ^1^, Alina Nussbaumer-Pröll^1^, Christine Radtke^5^, Markus Zeitlinger^1^, Felix Bergmann^1,5,*^

^1^Department of Clinical Pharmacology, Medical University of Vienna, Vienna, Austria

^2^IT Systems and Communications, Medical University of Vienna, Vienna Austria

^3^Division of Infectious Diseases and Tropical Medicine, Department of Medicine I, Medical University of Vienna, Vienna, Austria

^4^Department of Biomedical Imaging and Image-guided Therapy, Medical University of Vienna, Vienna, Austria

^5^Department of Plastic, Reconstructive and Aesthetic Surgery, Medical University of Vienna, Vienna, Austria

***Corresponding Author**

Felix.bergmann@meduniwien.ac.at

Department of Clinical Pharmacology, Medical University of Vienna

Department of Plastic, Reconstructive and Aesthetic Surgery, Medical University of Vienna

Währinger Gürtel 18-20, 1090, Vienna, Austria

Phone +43 40400 29810

**Tables**

**Table S1** List of excluded pathogens with corresponding explanation

| **Bacterial Pathogen** | **Exclusion criteria** |
| --- | --- |
| Staphylococcus species | Physiological skin or oropharyngeal flora |
| Acinetobacter radioresistens | Physiological skin or oropharyngeal flora |
| Micrococcus species | Physiological skin or oropharyngeal flora |
| Lactobacillus species | Physiological flora of gastrointestinal tract, urogenital tract, respiratory tract or female genital tract |
| Sphingomonas paucimobilis | Clinically insignificant as assessed by the treating physician |
| Lautropia mirabilis | Unclear pathogenic potential |
| Sphingobacterium multivorum | Clinically insignificant as assessed by the treating physician |
| Prevotella species | Physiological flora of gastrointestinal tract, urogenital tract, respiratory tract or female genital tract |
| Gemella haemolysans | Physiological flora of gastrointestinal tract, urogenital tract, respiratory tract or female genital tract |
| Pasteurella multocida | Clinically insignificant as assessed by the treating physician |
| Acinetobacter bereziniae | Opportunistic pathogen |
| Staphylococcus epidermidis | Physiological skin or oropharyngeal flora |
| Pantoea agglomerans | Opportunistic pathogen |
| Cronobacter species | Opportunistic pathogen |
| Staphylococcus hominis | Physiological skin or oropharyngeal flora |
| Staphylococcus pettenkoferi | Physiological skin or oropharyngeal flora |
| Staphylococcus capitis | Physiological skin or oropharyngeal flora |
| Cutibacterium (Propionibacterium) acnes | Physiological skin or oropharyngeal flora |
| Staphylococcus haemolyticus | Physiological skin or oropharyngeal flora |
| Brevibacterium ravenspurgense | Physiological skin or oropharyngeal flora, Opportunistic pathogen |
| Kocuria rhizophila | Physiological skin or oropharyngeal flora, Opportunistic pathogen |
| Staphylococcus petrasii | Physiological skin or oropharyngeal flora, Opportunistic pathogen |
| Eggerthella lenta | Physiological flora of gastrointestinal tract, urogenital tract, respiratory tract or female genital tract |
| Staphylococcus caprae | Physiological skin or oropharyngeal flora |
| Staphylococcus warneri | Physiological skin or oropharyngeal flora, Opportunistic pathogen |
| Gemella morbillorum | Physiological skin or oropharyngeal flora |
| Actinotignum schaalii | Physiological flora of gastrointestinal tract, urogenital tract, respiratory tract or female genital tract |
| Staphylococcus saccharolyticus | Physiological skin or oropharyngeal flora |
| Lactobacillus brevis | Physiological flora of gastrointestinal tract, urogenital tract, respiratory tract or female genital tract |

**Table S2** Baseline characteristics and outcomes per Covid-19 variant

|  | **Overall** | **Wildtype** | **Alpha** | **Delta** | **unknown** | **p** |
| --- | --- | --- | --- | --- | --- | --- |
| **n** | 1157 | 192 | 671 | 245 | 49 |  |
| **Length of stay, days, mean (SD)** | 16.41 (21.41) | 17.64 (26.74) | 16.32 (20.48) | 14.14 (15.32) | 24.31 (32.35) | 0.018 |
| **Male sex, n (%)** | 612 (52.9) | 112 (58.3) | 354 (52.8) | 126 (51.4) | 20 (40.8) | 0.147 |
| **Age, mean (SD)** | 58.92 (19.34) | 57.07 (20.00) | 61.11 (19.04) | 56.53 (18.75) | 48.16 (18.37) | <0.001 |
| **28-day mortality, n (%)** | 123 (10.6) | 16 (8.3) | 79 (11.8) | 25 (10.2) | 3 (6.1) | 0.379 |
| **Diabetes, n (%)** | 210 (18.2) | 36 (18.8) | 129 (19.2) | 39 (15.9) | 6 (12.2) | 0.468 |
| **Coronary artery disease, n (%)** | 187 (16.2) | 36 (18.8) | 104 (15.5) | 39 (15.9) | 8 (16.3) | 0.758 |
| **Chronic heart failure, n (%)** | 50 (4.3) | 9 (4.7) | 30 (4.5) | 11 (4.5) | 0 (0.0) | 0.507 |
| **Asthma, n (%)** | 25 (2.2) | 3 (1.6) | 13 (1.9) | 8 (3.3) | 1 (2.0) | 0.593 |
| **COPD, n (%)** | 76 (6.6) | 11 (5.7) | 41 (6.1) | 20 (8.2) | 4 (8.2) | 0.644 |
| **Chronic kidney disease, n (%)** | 128 (11.1) | 25 (13.0) | 70 (10.4) | 27 (11.0) | 6 (12.2) | 0.780 |
| **Skin disorder, n (%)** | 105 (9.1) | 19 (9.9) | 51 (7.6) | 29 (11.8) | 6 (12.2) | 0.188 |
| **Mental disorder, n (%)** | 238 (20.6) | 50 (26.0) | 128 (19.1) | 46 (18.8) | 14 (28.6) | 0.077 |
| **Neurologic disorder, n (%)** | 202 (17.5) | 30 (15.6) | 112 (16.7) | 49 (20.0) | 11 (22.4) | 0.446 |
| **Community-acquired infection, n (%)** | 35 (3.0) | 3 (1.6) | 17 (2.5) | 14 (5.7) | 1 (2.0) | 0.043 |
| **Early respiratory infection, n (%)** | 34 (2.9) | 3 (1.6) | 16 (2.4) | 14 (5.7) | 1 (2.0) | 0.033 |
| **Early blood infection, n (%)** | 2 (0.2) | 0 (0.0) | 1 (0.1) | 1 (0.4) | 0 (0.0) | 0.747 |
| **Hospital-acquired infection, n (%)** | 95 (8.2) | 20 (10.4) | 43 (6.4) | 25 (10.2) | 7 (14.3) | 0.050 |
| **Late respiratory infection, n (%)** | 93 (8.0) | 20 (10.4) | 41 (6.1) | 25 (10.2) | 7 (14.3) | 0.029 |
| **Late blood infection, n (%)** | 6 (0.5) | 0 (0.0) | 5 (0.7) | 1 (0.4) | 0 (0.0) | 0.576 |
| **Early test available, n (%)** | 357 (30.9) | 80 (41.7) | 197 (29.4) | 68 (27.8) | 12 (24.5) | 0.004 |
| **Late test available, n (%)** | 323 (27.9) | 57 (29.7) | 189 (28.2) | 60 (24.5) | 17 (34.7) | 0.412 |

**Figures**

**Figure S1** Hospital admissions of Covid-19 and Influenza patients over time

**Figure S2** Influenza variants over time

**Figure S3** Covid-19 variants over time

**Figure S4** 28-day mortality in the matched cohort (360 Covid-19 cases vs 180 Influenza cases)

**Figure S5** 28-day mortality in the overall (pre-matching) cohort (1157 Covid-19 cases vs 180 Influenza cases)

**Figure S6** CRP levels (mg/dL) in patients with and without bacterial co-infections in (A) the overall cohort, (B) in Covid-19 patients, and (C) in influenza patients.

**Figure S7** Procalcitonin levels (ng/mL) in patients with and without bacterial co-infections in (A) the overall cohort, (B) in Covid-19 patients, and (C) in influenza patients.

**Figure S8** Absolute frequency of early microbiological testing **(A and B)**, community-acquired bacterial co-infections **(C and D)**, late microbiological testing **(E and F)**, and hospital-acquired bacterial co-infections **(G and H)** in the Influenza (n=180) and Covid-19 (n=1157) group over time. To visual trends over time, we divided both groups separately into four equally large time periods containing 45 Influenza patients or 289 Covid-19 patients, respectively.

**Figure S9** All-cause 28-day mortality **(A and B)** and 28-day ICU admission **(C and D)** in the Influenza (n=180) and Covid-19 (n=1157) group over time.

To visual trends over time, we divided both groups separately into four equally large time periods containing 45 Influenza patients or 289 Covid-19 patients, respectively.
